# S-Adenosyl-L-Methionine for the Treatment of Chronic Liver Disease: A Systematic Review and Meta-Analysis

**DOI:** 10.1371/journal.pone.0122124

**Published:** 2015-03-16

**Authors:** Tao Guo, Lei Chang, Yusha Xiao, Quanyan Liu

**Affiliations:** Department of General Surgery, Research Center of Digestive Diseases, Zhongnan Hospital, Wuhan University, Wuhan 430071, P.R. China; University Hospital Oldenburg, GERMANY

## Abstract

It has been well established that S-adenosyl-L-methionine (SAMe) is the principal methyl donor in methyltransferase reactions and that SAMe supplementation restores hepatic glutathione (GSH) deposits and attenuates liver injury. However, the effectiveness of SAMe therapy in chronic liver disease has not been adequately addressed. We searched globally recognized electronic databases, including PubMed, the Cochrane Database and EMBASE, to retrieve relevant randomized controlled trials (RCTs) of chronic liver disease published in the past 20 years. We then performed a systematic review and meta-analysis of the enrolled trials that met the inclusion criteria.The results showed that twelve RCTs from 11 studies, which examined 705 patients, were included in this research. For liver function, certain results obtained from data synthesis and independent comparisons demonstrated significant differences between the levels of total bilirubin (TBIL) and aspartate transaminase (AST). However, no studies identified significant differences regarding alanine transaminase (ALT) levels. An analysis of the adverse events and long-term prognosis also indicated no significant differences between the SAMe and the placebo groups. In a subgroup analysis of gravidas and children, several of the included data indicated that there was a significant difference in the pruritus score. Furthermore, the results regarding ursodeoxycholic acid (UDCA) and stronger neo-minophagen C (SNMC) indicated that both treatments were more effective than SAMe was in certain chronic liver diseases. These findings suggest that SAMe could be used as the basis of a medication regimen for liver function improvement because of its safety. However, SAMe also demonstrated limited clinical value in the treatment of certain chronic liver diseases.

## INTRODUCTION

S-adenosyl-L-methionine (SAMe) is a pleiotropic molecule that is involved in multiple cellular reactions. This molecule participates in the following three types of reactions: transmethylation, transsulfuration and aminopropylation [1, 2]. It has been well established that SAMe is the principal methyl donor in methyltransferase reactions [3] and that SAMe supplementation restores hepatic glutathione (GSH) deposits and attenuates liver injury [4, 5]. SAMe is also involved in many biochemical reactions in the human body, serving as a key metabolite that regulates hepatocyte growth, death and differentiation [6]. In mammals, two genes (*MAT1A* and *MAT2A*) encode homologous MAT catalytic subunits [7, 8]. However, SAMe biosynthesis is depressed in patients with chronic liver disease [9]. Preclinical studies indicate that this depression might exacerbate liver injury; therefore, supplementation might represent a useful therapy [10].

In animal models, the relationship between intrahepatic SAMe depletion and hepatic fibrosis has been confirmed [11, 12]. In vitro experiments demonstrated that SAMe increases the antiviral effect of interferon; therefore, SAMe is considered as the first esoteric interferon sensitizer [13]. In the 1970s, SAMe was used as an anti-inflammatory analgesic for the treatment of arthritis and depression. Recently, SAMe could be also used as a safe and effective drug to reduce jaundice, especially for Chronic Hepatits B Patients with jaundice [14, 15]. However, similar studies that used animal models demonstrated that SAMe could not ameliorate liver cell necrosis and fibrosis [16, 17]. Based on research regarding the viral response, SAMe might be related to an early viral response, although SAMe does not induce a sustained viral response [18]. In recent years, an increasing number of patients with chronic liver disease have been treated with SAMe in different countries. As a relatively new method for the treatment of liver disease, a large number of clinical trials focusing on SAMe have commenced. However, a debate regarding SAMe has been ongoing for years; the results published in different nations in the past 20 years have indicated that there is no quantitative evidence, in the form of a comprehensive data analysis, for the efficacy of the treatment of chronic liver diseases with SAMe. This paper is thus the first systematic review and quantitative analysis of the effectiveness and safety of SAMe in the treatment of chronic liver diseases based on published randomized controlled trials (RCTs).

## METHODS

### Description of design

In recent years, many RCTs from different countries have been published. Therefore, the data could be searched for in native databases. For inclusion in this study, research had to have originated from studies that could be found in globally recognized databases. The studies were not limited to certain languages, although an English-language abstract had to be available for each study.

Comparisons of major biochemical parameters, such as the levels of total bilirubin (TBIL), alanine aminotransferase (ALT) and aspartate transaminase (AST), were analyzed to determine liver function. Additionally, the number of adverse events and the rate of death or liver transplants were identified to examine safety and long-term prognosis.

A subgroup analysis of gravidas and children was also performed in this research. Associated data or parameters were added to the proper analyses, such as the pruritus score for gravidas.

Because they are controversial drugs, an analysis of the clinical efficacy of ursodeoxycholic acid (UDCA) and stronger neo-minophagen C (SNMC) compared with SAMe in certain liver diseases was conducted as an additional analysis. In particular, data isolation and extraction from the studies of UDCA or SNMC were conducted, and all of the related parameters were considered, if possible.

The final results of this research are described using quantitative analysis and subjective evaluations. The specific chronic liver diseases examined in this study included chronic hepatitis, cirrhosis, cholestasis, hepatic adipose infiltration and alcoholic liver disease. Chronic drug-induced liver disease, liver cancer and post-hepatectomy patients were also included.

### Data sources and searches

We searched the Cochrane, PubMed and EMBASE databases for articles published from May 1994 to May 2014, using the following criteria(S1 Table): ((chronic liver disease[All Fields] OR liver function[All Fields]) AND S-adenosyl-L-methionine [All Fields]). A secondary review of the included articles and their references and presentations from the 2 largest US gastroenterology/hepatology research meetings, the AASLD Liver Meeting (from 2010 to 2013) and Digestive Disease Week (from 2010 to 2013) was conducted using the search term “chronic liver disease” and reviewed for possible inclusion. We manually searched the reference lists of the reports of trials included in the review for additional trials. We used the Science Citation Index to find studies that had cited the included trials. We included all germane original English language studies of human research and missing data were manually searched or were requested from the authors.

The inclusion criteria included the following: (1) case-controlled and randomized trials; (2) related parameters should be provided in the studies; (3) raw data for liver function should include the mean and standard deviation; (4) the patients should have been clearly diagnosed as having correlative chronic liver disease, without severe complications; and (5) patients in the same group should have been treated equally during the trial.

The exclusion criteria eliminated studies with the following characteristics(S2 Table): (1) no control group, (2) incomplete raw data for the purposes of this research, (3) limitation to animals or cells, (4) a publication date prior to 1994, and (5) levels of the liver function parameters prior to treatment that were different between the experimental and the control groups.

### Standards of literature quality assessment

We judged the risk of bias according to the criteria outlined in the Cochrane Handbook for Systematic Reviews [19]. Studies were included if they met all of the following criteria: (1) free of selection bias, (2) free of performance bias, (3) free of detection bias, 4) free of attrition bias, (5) free of reporting bias, and (6) free of other bias.

Each selected study was required to be judged based on these criteria. We subsequently performed a comprehensive examination regarding the quality of all of the included studies.

### Data extraction and analysis

The data extraction was independently accomplished by two authors (Tao Guo and Lei Chang). Any disputes were resolved through discussion or were decided on by a third author (Quanyan Liu). According to the design, the data regarding liver function, which were considered as continuous variables in different trials, were analyzed separately because of the different baseline values (the levels of the respective parameters prior to treatment). However, data synthesis was also conducted if the respective baseline values from the different trials exhibited no significant differences after an unpaired t-test. The data regarding changes in these parameters could also be directly compared. Comparisons were made between the SAMe and the placebo groups or between the SAMe plus UDCA (or SNMC) and the UDCA (or SNMC)-alone groups. However, the data regarding dichotomous variables were comprehensively compared by ignoring the different backgrounds.

In a subgroup analysis of gravidas and children, a comparison of pruritus scores was performed.

In an additional analysis, all of the data were analyzed to evaluate the efficacy and safety of specific interventions compared with SAMe. The groups were compared as previously described.

RevMan5.0 software, provided by The Cochrane Library, was used for the data synthesis and comparisons. Heterogeneity (I^2^ index statistic) in the study design was used to estimate a data model. We estimated the pooled estimates of the risk ratio and mean difference. The relevant 95% confidence interval (CI) that used fixed (I^2^<50%) or random (I^2^>50%) effects models depended on the heterogeneity of the included trials [20, 21].

## RESULTS

**Fig 1:**
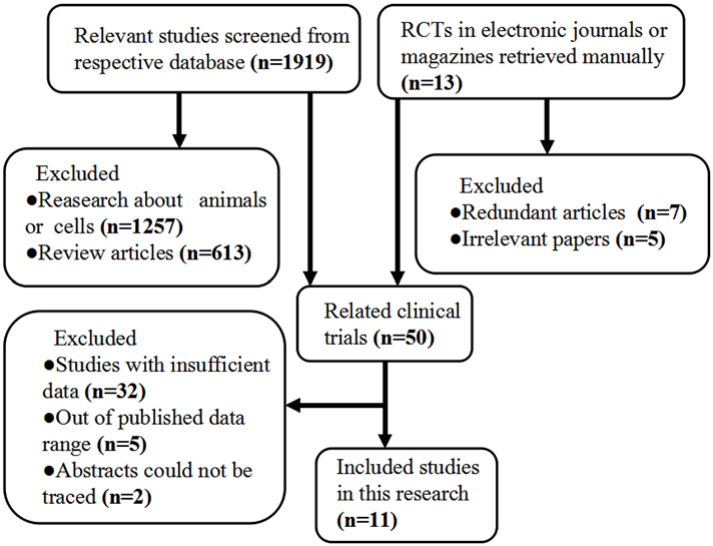
Flow diagram of the process (and the reasons) of selecting and excluding studies for this meta-analysis.

A flow chart of the selection of the included trials is given in Fig. 1 and S1 Fig. 11 studies [22–32] that included RCTs were identified in the present analysis. Two studies were excluded because they can not be located, one study published in Spanish and another study published in Russian. The 11 included studies involved 705 patients with 7 types of chronic liver diseases.

### Characteristics and data processing

The entities, interventions, doses, treatment times, and available parameters were included in the data extraction process. Certain descriptions of the main items are introduced in the subsequent sections, and a summary of the characteristics of the included studies is provided in Table 1.

**Table 1:** Characteristics of the included trials.

Author	Time of pub.	Country	Related disease	Intervention	SAMe dose	Time of treatment	Available parameter	Remark
*P*. *L*. *Nicastri*	1998	Italy	Gestational cholestasis	SAMe versus vitamin/SAMe plus UDCA versus UDCA alone/SAMe versus UDCA	800 mg/d	20 days	ALT/TBIL/pruritus score	Gravidas
*Huang Jinyang*	2004	China	Gestational cholestasis	SAMe versus Yingchenghao decoction	1000 mg/d	3 weeks	ALT/TBIL/pruritus score	Gravidas
*Zhu Shishu*	2010	China	Drug-induced liver disease	SAMe versus Yinzhihuang granule plus compound glycyrrhizin	20–30 mg/kg/d	4 weeks	ALT/AST/TBIL	Children
*Qin Bo*	2000	China	Cholestatic hepatitis/viral hepatitis with cholestasis	SAMe versus potassium magnesium aspartate	1000 mg/d	4 weeks	ALT/AST/TBIL	—
*Sun Qingfeng*	2010	China	Hepatitis B virus	SAMe versus SNMC	1000 mg/d	4 weeks	ALT/AST/number of adverse events	Gravidas
*Jose M*. *Mato*	1999	Italy	Alcoholic liver cirrhosis	Not mentioned	1200 mg/d	2 years	Number of deaths or liver transplants	—
*T*. *Binder*	2006	Czech	Gestational cholestasis	SAMe plus UDCA versus UDCA alone/SAMe versus UDCA	1000 mg/d	4 weeks	ALT/AST/TBIL/number of adverse events	Gravidas
*Valentina Medici*	2011	USA	Alcoholicliver disease	Not mentioned	1200 mg/d	24 weeks	Number of adverse events	—
*Su Zhaoran*	2013	China	Post-hepatectomy	Not mentioned	1000 mg/d	7 days	Number of adverse events	—
*Nadia Roncaglia*	2004	Italy	Gestational cholestasis	SAMe versus UDCA	1000 mg/d	4 weeks	Pruritus score/number of deaths	Gravidas
*V*. *V*. *Stelmakh*	2013	Russia	Nonalcoholic fatty liver disease	Not mentioned	400 mg/d	11 days	TBIL	—

#### Entities

We included 7 types of chronic liver diseases from 5 different nations. A trial by *Qin Bo* regarding acute hepatitis with cholestasis was excluded. *P*. *L*. *Nicastri*, *Huang Jinyang*, *T*. *Binder* and *Nadia Roncaglia* reported gestational cholestasis. Alcoholic liver diseases were presented in separate studies by *Jose M*. *Mato* and *Valentina Medici*. *Qin Bo* and *Sun Qingfeng* provided disease data regarding viral hepatitis. Drug-induced liver disease, post-hepatectomy patients, nonalcoholic fatty liver disease and cholestatic hepatitis were described by *Zhu Shishu*, *Su Zhaoran*, *V*. *V*. *Stelmakh* and *Qin Bo*. Five studies regarding gravidas originated from *P*. *L*. *Nicastri*, *Huang Jinyang*, *Sun Qingfeng*, *T*. *Binder* and *Nadia Roncaglia*. Finally, 1 study focusing on children was published by *Zhu Shishu*.

#### Interventions

We considered UDCA and SNMC as intervention drugs, and all other drugs were considered as normal placebos. UDCA was described by *P*. *L*. *Nicastri*, *T*. *Binder* and *Nadia Roncaglia*. SNMC was only analyzed by *Sun Qingfeng*. In contrast, *Jose M*. *Mato*, *Valentina Medici*, *Su Zhaoran* and *V*. *V*. *Stelmakh* did not describe the details of the placebos used. Therefore, we assumed that these studies utilized normal placebos, similar to the placebos used by *Jose M*. *Mato*, *Valentina Medici*, *Su Zhaoran* and *V*. *V*. *Stelmakh*.

Doses and treatment times: Regarding the timing of therapy, the trial by *Zhu Shishu* indicated a dose of 20–30 mg/kg/d for children. *V*. *V*. *Stelmakh* provided data in a study using 400 mg/d, which was clearly lower than the dose used in other trials. The remaining studies utilized doses of 800–1200 mg/d, which was considered as the baseline. Meanwhile, *Jose M*. *Mato* and *Valentina Medici* each examined the long-term effects of alcoholic liver diseases (>3 months), whereas the other studies reported various courses of treatment that were appropriate for the respective diseases (<3 months).

#### Parameter selection

At least one available parameter in each study was one of the inclusion criteria. Baseline verifications between groups in every trial were performed when we extracted the TBIL, ALT and AST data. Certain parameters that included a mean value without a standard deviation were excluded, such as the TBIL, ALT and AST data from the study by *Valentina Medici*, and all of the measurement units were unified at the same time.

### Quality evaluation

**Fig 2:**
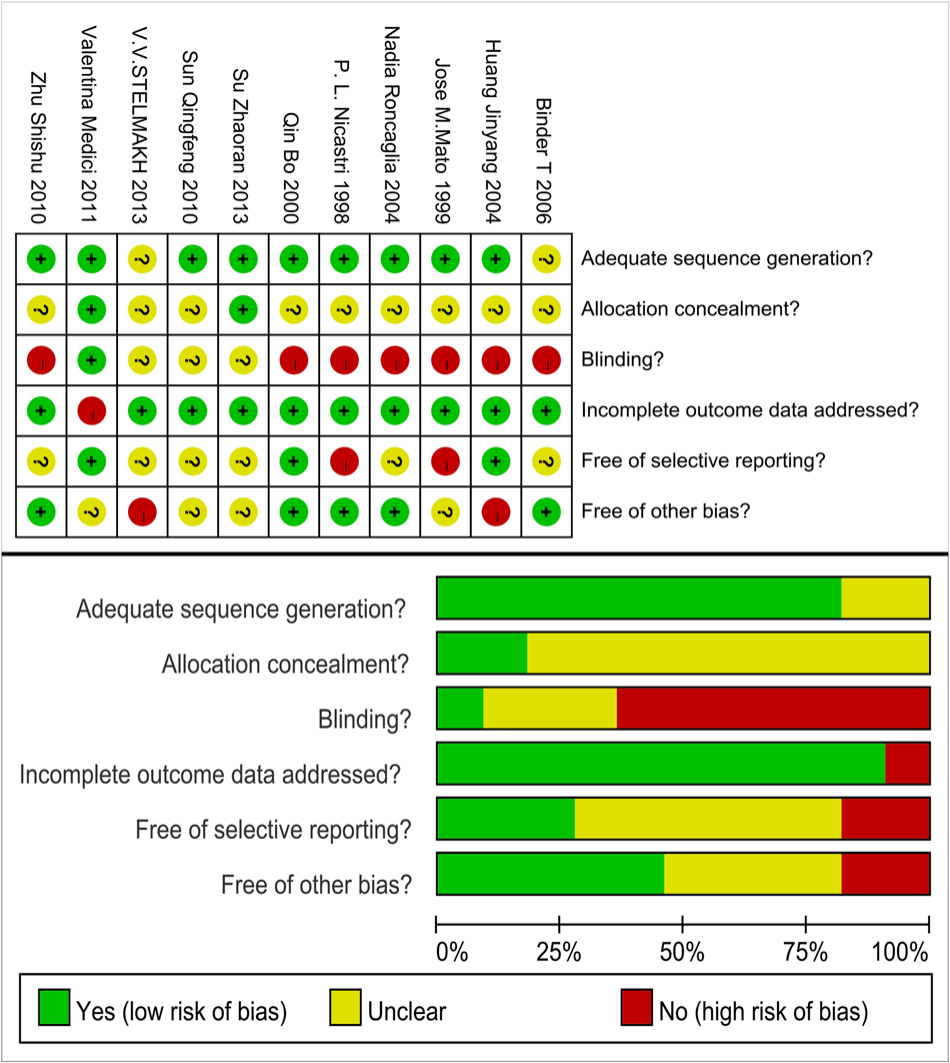
Methodological quality graph and summary of the included studies.

All of the studies were randomized, with the exception of the studies by *T*. *Binder* and *V*. *V*. *Stelmakh*; the design methods of these two studies were not described. Only *Valentina Medici* and *Su Zhaoran* provided their allocation concealments; the other researchers did not report this information. Additionally, blinding of both the participants and the investigators was only reported by *Valentina Medici*. In contrast, the blinding scheme was unclear in the studies by *Su Zhaoran*, *Sun Qingfeng* and *V*. *V*. *Stelmakh*. However, we were certain that there was no blinding in the remaining studies. Eleven dropouts were identified in the study by *Valentina Medici*, whereas no outcome data were missing in the other studies. Although only one available parameter was provided in certain studies included in the present research, most of the included studies were comprehensive. The imbalance in the numbers of participants between the experimental and the control groups (such as in the studies by *Huang Jinyang* and *V*. *V*. *Stelmakh*) and different research directions (such as in the studies by *Sun Qingfeng* and *Jose M*. *Mato*) might have resulted in potential bias in this research. Based on this assessment, we concluded that these findings could be included in the present research, despite a potential unidentified bias. A summary of the bias risk is shown in Fig. 2.

### SAMe decreased the TBIL and AST levels but did not improve the ALT levels in patients with chronic liver diseases

After comparing the parameters based on the methods previously described, we obtained results regarding liver function, adverse events and long-term prognosis. We also obtained results from the subgroup and additional analyses.

**Fig 3:**
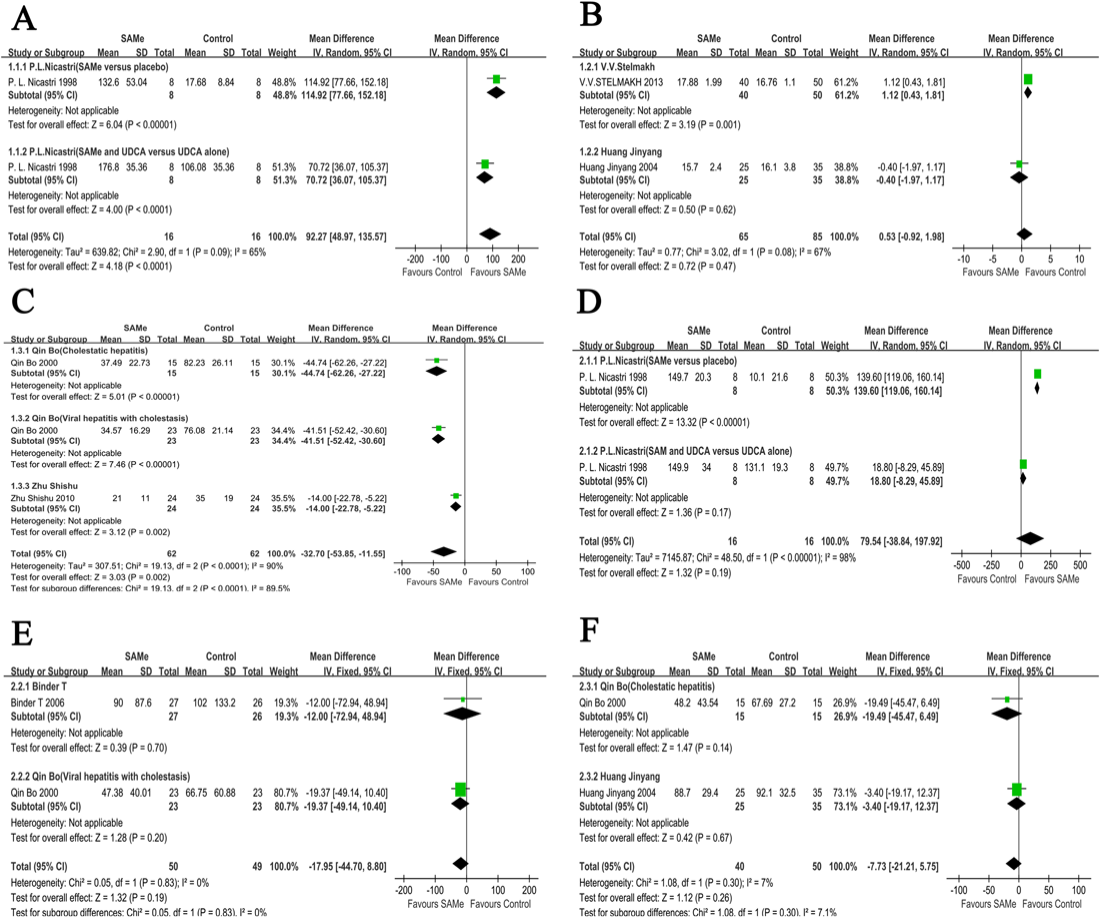
Meta-analysis of TBIL and ALT levels. **(A)** Meta-analysis of TBIL levels based on the study by *P*. *L*. *Nicastri*. **(B)** Meta-analysis of TBIL levels based on the studies by *Huang Jinyang* and *V*. *V*. *Stelmakh*. **(C)** Meta-analysis of TBIL levels based on the studies by *Qin Bo* and *Zhu Shishu*. **(D)** Meta-analysis of ALT levels based on the study by *P*. *L*. *Nicastri*. **(E)** Meta-analysis of ALT levels based on the studies by *Qin Bo* and *T*. *Binder*. **(F)** Meta-analysis of ALT levels based on the studies by *Qin Bo* and *Huang Jinyang*.

There were 8 independent comparisons for the TBIL levels from 6 studies, which included 359 participants. The relevant data are presented in Table 2. Based on the data, we performed a data synthesis for the TBIL levels (Fig. 3A, 3B and 3C) from the 8 independent comparisons (Table 2). After the data synthesis, two comparisons between the SAMe and the placebo groups exhibited statistically significant results (MD [95% CI] = 92.27 [48.97, 135.57], P<0.0001; MD [95% CI] = -32.7 [-53.85,-11.55], P = 0.002) (Fig. 3A and 3C).

**Table 2:** Data and comparisons regarding the included parameters.

Parameter	Author	Related disease	Intervention	Data type	Baseline (μmol/L) & (U/L)		No. of patients	Statistical method	Effect size	P value	Data synthesis*
					SAMe	Control					
TBIL	*P*. *L*. *Nicastri*	Intrahepatic cholestasis	SAMe versus placebo	Values of changes after treatment	—	—	16	Inverse variance	114.92 [77.66,152.18]	P<0.05	{1}
TBIL	*P*. *L*. *Nicastri*	Intrahepatic cholestasis	SAMe and UDCA versus UDCA alone	Values of changes after treatment	—	—	16	Inverse variance	70.72 [36.07,105.37]	P<0.05	{1}
TBIL	*V*. *V*. *Stelmakh*	Nonalcoholic fatty liver disease	SAMe versus placebo	Baseline and final values after treatment	26.56±1.53	24.38±1.38	90	Inverse variance	1.12 [0.43,1.81]	P<0.05	{2}
TBIL	*Huang Jinyang*	Intrahepatic cholestasis	SAMe versus placebo	Baseline and final values after treatment	27.9±5.1	28.3±4.2	60	Inverse variance	-0.40 [-1.97,1.17]	P = 0.62	{2}
TBIL	*T*. *Binder*	Intrahepatic cholestasis	SAMe and UDCA versus UDCA alone	Baseline and final values after treatment	18±6.9	15.1±7.79	53	Inverse variance	0.80 [-1.49,3.09]	P = 0.49	—
TBIL	*Qin Bo*	Cholestatic hepatitis	SAMe versus placebo	Baseline and final values after treatment	171.97±59.42	152.56±49.83	30	Inverse variance	-44.74 [-62.26,-27.22]	P<0.05	{3}
TBIL	*Qin Bo*	Viral hepatitis with cholestasis	SAMe versus placebo	Baseline and final values after treatment	218.01±118.58	205.66±97.37	46	Inverse variance	-41.51 [-52.42,-30.60]	P<0.05	{3}
TBIL	*Zhu Shishu*	Drug-induced liver disease	SAMe versus placebo	Baseline and final values after treatment	197±69	198±68	48	Inverse variance	-14.00 [-22.78,-5.22]	P<0.05	{3}
ALT	*P*. *L*. *Nicastri*	Intrahepatic cholestasis	SAMe versus placebo	Values of changes after treatment	—	—	16	Inverse variance	139.60 [119.06,160.14]	P<0.05	{4}
ALT	*P*. *L*. *Nicastri*	Intrahepatic cholestasis	SAMe and UDCA versus UDCA alone	Values of changes after treatment	—	—	16	Inverse variance	18.80 [-8.29,45.89]	P = 0.17	{4}
ALT	*Huang Jinyang*	Intrahepatic cholestasis	SAMe versus placebo	Baseline and final values after treatment	180.5±22.6	175.4±25.3	60	Inverse variance	-12.00 [-72.94,48.94]	P = 0.70	{5}
ALT	*T*. *Binder*	Intrahepatic cholestasis	SAMe and UDCA versus UDCA alone	Baseline and final values after treatment	384±246	282±229.2	53	Inverse variance	-19.37 [-49.14,10.40]	P = 0.20	{6}
ALT	*Qin Bo*	Cholestatic hepatitis	SAMe versus placebo	Baseline and final values after treatment	198.47±75.20	190.61±71.24	30	Inverse variance	-19.49 [-45.57,6.49]	P = 0.14	{5}
ALT	*Qin Bo*	Viral hepatitis with cholestasis	SAMe versus placebo	Baseline and final values after treatment	280.96±214.83	284.75±237.58	46	Inverse variance	-3.40 [-19.17,12.37]	P = 0.67	{6}
ALT	*Zhu Shishu*	Drug-induced liver disease	SAMe versus placebo	Baseline and final values after treatment	488±248	485±256	48	Inverse variance	0.00 [-10.50,10.50]	P = 1.00	—
AST	*T*. *Binder*	Intrahepatic cholestasis	SAMe and UDCA versus UDCA alone	Baseline and final values after treatment	249±111	198±106.8	53	Inverse variance	-16.00 [-25.33,-6.67]	P<0.05	—
AST	*Qin Bo*	Cholestatic hepatitis	SAMe versus placebo	Baseline and final values after treatment	127.04±60.65	118.15±58.70	30	Inverse variance	-17.37 [-43.73,8.99]	P = 0.20	—
AST	*Qin Bo*	Viral hepatitis with cholestasis	SAMe versus placebo	Baseline and final values after treatment	262.19±250.80	267.25±230.42	46	Inverse variance	-7.13 [-26.29,12.03]	P = 0.47	{7}
AST	*Zhu Shishu*	Drug-induced liver disease	SAMe versus placebo	Baseline and final values after treatment	311±163	306±164	48	Inverse variance	9.00 [-27.06,45.06]	P = 0.62	{7}
Adverse events	*T*. *Binder*	Intrahepatic cholestasis	SAMe and UDCA versus UDCA alone	Number	—	—	53	Mantel-Haenszel	2.89 [0.32,26.02]	P = 0.34	{8}
Adverse events	*Su Zhaoran*	Post-hepatectomy	SAMe versus placebo	Number	—	—	79	Mantel-Haenszel	0.72 [0.40,1.31]	P = 0.29	{8}
Adverse events	*Valentina Medici*	Alcoholicliver disease	SAMe versus placebo	Number	—	—	37	Mantel-Haenszel	1.23 [0.51,2.97]	P = 0.64	{8}
Ratio of death	*Jose M*. *Mato*	Alcoholic liver cirrhosis	SAMe versus placebo	Number	—	—	123	Mantel-Haenszel	0.55 [0.27,1.09]	P = 0.09	—
Pruritus score	*P*. *L*. *Nicastri*	Intrahepatic cholestasis	SAMe versus placebo	Values of changes after treatment	—	—	16	Inverse variance	0.50 [0.35,0.65]	P<0.05	{9}
Pruritus score	*P*. *L*. *Nicastri*	Intrahepatic cholestasis	SAMe and UDCA versus UDCA alone	Values of changes after treatment	—	—	16	Inverse variance	1.00 [0.60,1.40]	P<0.05	{9}
Pruritus score	*Huang Jinyang*	Intrahepatic cholestasis	SAMe versus placebo	Baseline and final values after treatment	3.5±0.6	3.6±0.6	60	Inverse variance	0.10 [-0.12,0.32]	P = 0.37	—

*Marking with the same number “{x}” indicates data synthesis in the same group.

For the ALT levels, there were 7 independent comparisons from 6 studies, which included 269 patients. The relevant data are presented in Table 2. For the final results, the data synthesis is provided according to the data types and baselines. However, none of the analyses identified significant differences (MD [95% CI] = 79.54 [-38.84, 197.92], P = 0.19; MD [95% CI] = -17.95 [-44.70, 8.80], P = 0.19; MD [95% CI] = -7.73 [-21.21, 5.75], P = 0.26) (Fig. 3D, 3E and 3F).

**Fig 4:**
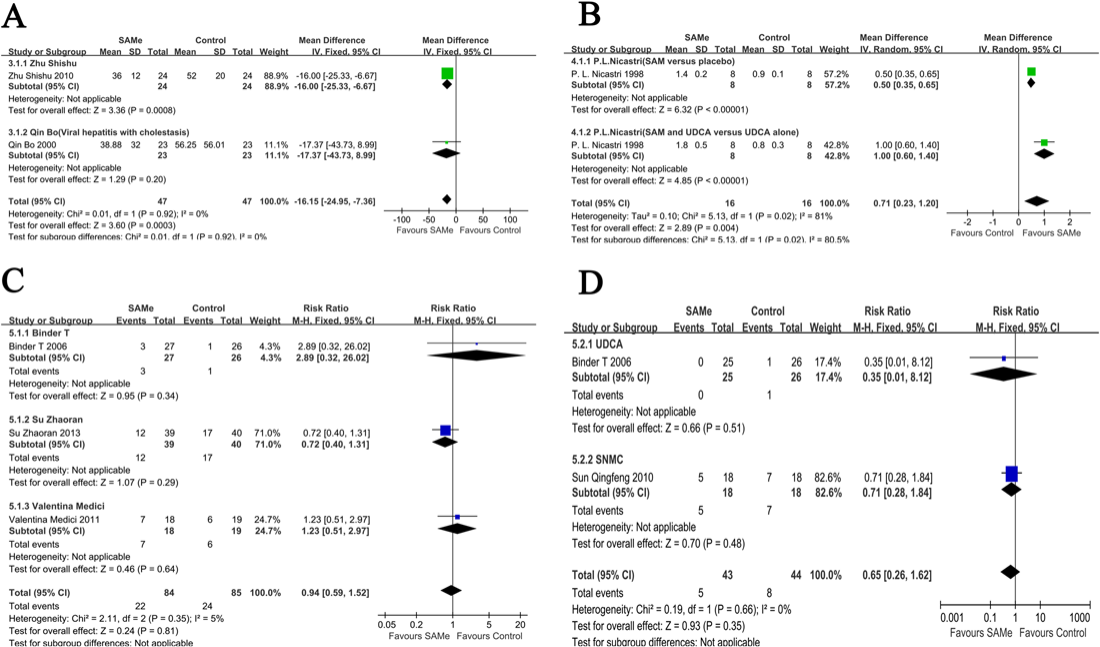
Data analysis and synthesis of the AST levels, pruritus scores and numbers of adverse events based on respective baselines or data types. **(A)** Meta-analysis of AST levels based on the studies by *Qin Bo* and *Zhu Shishu*. **(B)** Meta-analysis of pruritus scores in the subgroup analysis. **(C)** Meta-analysis of adverse events for SAMe compared with placebos. **(D)** Meta-analysis of adverse events in an additional analysis.

There were 4 independent comparisons of the AST levels from 3 studies, which included 177 patients. The related data are shown in the Table 2. Based on the respective baseline values, a data synthesis was conducted. The results of the data synthesis were significantly different (MD [95% CI] = -16.15 [-24.95,-7.36], P = 0.0003) (Fig. 4A).

### SAMe did not increase the number of adverse events or the death rate compared with the placebo

There were 3 trials (the trial of SAMe compared with UDCA from *T*. *Binder* is not included here), including 169 patients, that provided the numbers of adverse events. The results indicated that there was no significant difference between the SAMe and the placebo groups (RR [95% CI] = 0.94 [0.59, 1.52], P = 0.81) (Fig. 4C).

Only one study, which included 123 patients, provided the rates of death and liver transplants. The results were used to assess the long-term prognosis of SAMe treatment. However, there was no significant difference between the SAMe and the placebo groups (OR [95% CI] = 0.55 [0.27, 1.09], P = 0.09) (Table 2).

### Subgroup analysis of gravidas and children

There were 3 gravidas studies and 1 child study (SAMe compared with UDCA or SNMC was not included). *P*. *L*. *Nicastri*, *Huang Jinyang* and *T*. *Binder* included gravidas, and the study by *Zhu Shishu* included children.

In the gravidas subgroup, parameters such as the TBIL, ALT, and AST levels and the number of adverse events were considered. In addition, data regarding pruritus scores were included for all 193 patients (Table 2). For the TBIL levels, only *P*. *L*. *Nicastri* identified a significant difference between the SAMe and the placebo groups (MD [95% CI] = 92.27 [48.97, 135.57], P<0.0001). For the ALT levels, none of the comparisons in the included studies identified statistically significant results. Finally, for the AST levels, only *T*. *Binder* provided a comparison that demonstrated that there was no statistically significant difference (MD [95% CI] = 9.00 [-27.06, 45.06], P = 0.62). Additionally, for adverse events, only *T*. *Binder* provided data, and the results indicated that there was no statistically significant difference (RR [95% CI] = 2.89 [0.32, 26.02], P = 0.34). We provide the data regarding pruritus scores in Table 2. The results of the data synthesis from *P*. *L*. *Nicastri* identified an obvious difference (MD [95% CI] = 0.71 [0.23, 1.20], P = 0.004) (Fig. 4B). In contrast, *Huang Jinyang* identified a difference without statistical significance (MD [95% CI] = 0.10 [-0.12, 0.32], P = 0.37) (Table 2).

Only *Zhu Shishu* provided findings regarding children; this author’s study included 48 participants. We present comparisons regarding the levels of TBIL, ALT and AST in Table 2. We observed a significant difference in the TBIL levels (WD [95% CI] = -14 [-22.78,-5.22], P = 0.002) and AST levels (WD [95% CI] = -16 [-25.33,-6.67], P = 0.0008). However, no significant difference was identified for the ALT levels (WD [95% CI] = 0.00 [-10.5, 10.5], P = 1.00).

### Comparative analysis of the efficacy and safety of SAMe, UDCA and SNMC

Four studies that included 149 participants and focused on the effects of UDCA (or SNMC) compared with SAMe were analyzed. All of the related data and comparisons are presented in Table 3.

**Table 3:** Related data and comparisons for additional analysis.

Parameter	Author	Related disease	Intervention	Data type	Baseline (μmol/L) & (U/L)		No. of patients	Statistical method	Effect size	P value	Data synthesis*
					SAMe	Intervention					
TBIL	*P*. *L*. *Nicastri*	Intrahepatic cholestasis	SAMe versus UDCA	Values of changes after treatment	—	—	16	Inverse variance	26.52 [-17.65,70.69]	P = 0.24	—
TBIL	*T*. *Binder*	Intrahepatic cholestasis	SAMe versus UDCA	Baseline and final values after treatment	17.0±6.33	15.1±7.79	51	Inverse variance	4.65 [1.91,7.39]	P<0.05	—
ALT	*P*. *L*. *Nicastri*	Intrahepatic cholestasis	SAMe versus UDCA	Values of changes after treatment	—	—	16	Inverse variance	18.60 [-0.81,38.01]	P = 0.06	—
ALT	*T*. *Binder*	Intrahepatic cholestasis	SAMe versus UDCA	Baseline and final values after treatment	283.2±176.4	282±229.2	51	Inverse variance	132.00 [50.61,213.39]	P<0.05	—
ALT	*Sun Qingfeng*	Hepatitis B virus	SAMe versus SNMC	Baseline and final values after treatment	525.61±483.87	558.28±390.24	36	Inverse variance	70.36 [29.24,111.48]	P<0.05	—
AST	*T*. *Binder*	Intrahepatic cholestasis	SAMe versus UDCA	Baseline and final values after treatment	160.2±100.2	198±106.8	51	Inverse variance	108.60 [56.97,160.23]	P<0.05	—
AST	*Sun Qingfeng*	Hepatitis B virus	SAMe versus SNMC	Baseline and final values after treatment	510.78±621.58	558.28±390.24	36	Inverse variance	41.79 [11.30,72.28]	P<0.05	—
Pruritus score	*Nadia Roncaglia*	Gestational cholestasis	SAMe versus UDCA	Number	—	—	46	Mantel-Haenszel	1.01 [0.62,1.65]	P = 0.96	—
Adverse events	*T*. *Binder*	Intrahepatic cholestasis	SAMe versus UDCA	Number	—	—	51	Mantel-Haenszel	0.35 [0.01,8.12]	P = 0.51	{10}
Adverse events	*Sun Qingfeng*	Hepatitis B virus	SAMe versus SNMC	Number	—	—	36	Mantel-Haenszel	0.71 [0.28,1.84]	P = 0.48	{10}

*Marking with the same number “{x}” indicates data synthesis in the same group.

For UDCA compared with SAMe, separate studies from *P*. *L*. *Nicastri* and *T*. *Binder* focused on the TBIL levels. According to the data, 2 comparisons that were made served as the final results, and one comparison was significantly different (MD [95% CI] = 4.65 [1.91, 7.39], P = 0.0009). Regarding the ALT levels, 2 included studies provided data, and one of the 2 comparisons was significantly different (MD [95% CI] = 132.00 [50.61, 213.39], P = 0.001). Only one study provided data regarding UDCA compared with SAMe in terms of the AST levels, which exhibited a significant difference (MD [95% CI] = 108.60 [56.97,160.23], P<0.0001). Moreover, the study by *Nadia Roncaglia* found no statistically significant difference regarding pruritus score reductions in gravidas (RR [95% CI] = 1.01 [0.62, 1.65], P = 0.96).

For SNMC compared with SAMe, 1 comparison was significantly different regarding the ALT levels (MD [95% CI] = 70.36 [29.24, 111.48], P = 0.0008). One comparison also demonstrated a significant difference in the AST levels (MD [95% CI] = 41.79 [11.30, 72.28], P = 0.007).

For adverse events with UDCA and SNMC, a data synthesis was performed, but there was no significant difference (RR [95% CI] = 0.65 [0.26, 1.62], P = 0.35) (Fig. 4D).

## DISCUSSION

To the best of our knowledge, this paper is the first systematic review of quantitative analyses examining the use of SAMe in the treatment of chronic liver diseases of various etiologies. We detected associations between SAMe treatment and liver function parameters. The safety and long-term prognosis of SAMe treatment were also examined. Based on the literature published in the past 20 years, we identified 11 studies in globally recognized databases.

We found that the treatment of SAMe could improve liver function in chronic liver diseases, although there were no noticeable effects regarding reduction of the ALT levels according to these analysis results. However, reduction of the TBIL and AST levels was *significant*. The subgroup analysis results for gravidas and for children did not exhibit any unexpected shifts. Moreover, the effectiveness of SAMe in reducing the pruritus score was found basing on the results of the subgroup analysis. Therefore, we could also draw the same conclusion not only in adults but also in gravidas and children according to these findings. The conclusion was supported by the findings of previous trials based on pregnant women [33]. But, the study from a *previously* published meta-analysis probably doesn't support our observation well enough [34], which could not find evidence supporting or refuting the use of SAMe for patients with alcoholic liver diseases, and suggested that more long-term, high-quality randomised trials on SAMe for these patients should be completed before SAMe might be recommended for clinical practice. However, the study about the beneficial effects of SAMe for patients with alcoholic liver diseases mainly focus on liver-related mortality, all-cause mortality, liver transplantation, and complications in this meta-analysis. It did not analysis whether the treatment with SAMe could improve liver function in alcoholic liver diseases. And also there are a few updates about alcoholic liver diseases were published after that meta-analysis was published.

When the adverse events associated with the treatment of SAMe were analyzed, the results indicated that there was no significant difference between the SAMe and the placebo groups. These results support the findings of previous trials based on pregnant women [33] and also was verified by a previous meta-analysis [34], which showed that SAMe was not significantly associated with non-serious adverse events and no serious adverse events were reported.

UDCA can inhibit the absorption of hydrophobic bile acid in the ileum, and it is also involved in inhibition of the liver cell necrosis and apoptosis induced by the hydrophobic bile acid [35, 36]. UDCA has been used to treat cholestasis in recent years, as it can promote the secretion of bile via a protein kinase C-dependent pathway [37]. Meanwhile, SNMC plays a role as an interferon inducer, and it has been previously used to treat viral hepatitis. Additionally, SNMC is known to be an effective anti-inflammatory and cytoprotective agent [38]. In the current analysis, both drugs had clinical curative effects that were different from the effects of normal placebos. Therefore, the trials regarding UDCA (or SNMC) compared with SAMe were separated in the comparisons, and we analyzed their effects using additional analyses. The results regarding liver function provided evidence that both UDCA and SNMC had better efficacies than SAMe did in treating cholestasis and viral hepatitis. Because the results suggested that UDCA may affect gravidas, reductions in the pruritus score were analyzed; however, no significant differences were identified. In addition, no additional risk was associated with treatment with UDCA or SNMC.

Based on retrieval principles, English-language abstracts must be searched in globally recognized databases to avoid a geographical bias. Therefore, certain aspects of the data might have been insufficient, and we might have overlooked eligible studies. However, the results demonstrated several significant associations, even though because of the limitations of the data types, we were not able to gather all of the data in a comprehensive data synthesis. From the respective comparisons, we could observe the advantages of SAMe treatment in different types of chronic diseases, although the evidence was not supported by every single trial. In addition, there was only a single trial, by *V*. *V*. *Stelmakh*, that demonstrated that SAMe was less efficacious than the placebo was (Fig. 3B). Based on the accuracy of the data, we could also infer that the SAMe doses administered to the patients were clearly lower than in other studies. This finding might represent the key reason why adverse results were identified. It was also demonstrated that other treatment doses were utilized in other studies and that only one study investigated SNMC. In view of these studies, the effects of SAMe treatment in children might be predictable. Furthermore, the existing data regarding children and related research are expected.

For the first time, we examined the efficacy and safety of SAMe treatment for chronic liver diseases based on authoritative data. The influences of different liver diseases or patient characteristics must also be evaluated; we could then determine whether SAMe could be used as a first-line drug or as a basic medication. The lack of evidence regarding this type of data was most likely a major deficiency of the research presented here. Thus, the lack of evidence might have limited the conclusions that could be drawn. However, providing direction for correlative research was also important for our research aims.

The topic of SAMe treatment is not a new research field in liver disease. However, comprehensive discussions and qualitative analyses are not easy because of the limitations of the data provided and because of the linkage heterogeneity of the parameters. Due to this lack of related data, the correlation between SAMe treatment and other factors, such as entities, the placebo type, therapy times, and the long-term survival rate, could not be assessed. Consequently, certain inevitable bias might exist in the results. Given these factors, we are planning more relevant clinical research, and we will include new findings in future updates to the present research.

### Authors’ conclusions

Despite the absence of certain data and the existence of several limitations, our final conclusions support the efficacy and safety of SAMe for the treatment of chronic liver diseases. In particular, SAMe can help to improve liver function and, at the correct dosage, could be used as the basis of a medication regimen. But SAMe does not improve outcome or reduce the occurrence of adverse events for chronic liver diseases. And also, for certain diseases, such as cholestasis or virus hepatitis, UDCA and SNMC are each more effective than SAMe is. Therefore, the results presented here have limited clinical value. Additionally, because of the previously discussed issues, research regarding basic treatment with SAMe requires additional studies in the future. Studies of new drugs for the treatment of certain liver diseases might also represent a more valuable research direction.

## Supporting Information

S1 Fig Flow Diagram. 11 studies that included RCTs were identified in the present analysis. Two studies were excluded because they can not be located, one study published in Spanish and another study published in Russian. The 11 included studies involved 705 patients with 7 types of chronic liver diseases.
(JPG)

S1 PRISMA Checklist The PRISMA statement for reporting systematic reviews and meta-analyses. (DOC)

S1 Table The search strategy of PubMed database (XLS)

S2 Table The information of excluded RCTs (XLS)
