# Supplementary figures and images for: S-Adenosyl-L-Methionine for the Treatment of Chronic Liver Disease: A Systematic Review and Meta-Analysis
Source: PLoS One. 2015 Mar 16;10(3):e0122124. doi: 10.1371/journal.pone.0122124 (PMC4361566; doi:10.1371/journal.pone.0122124)

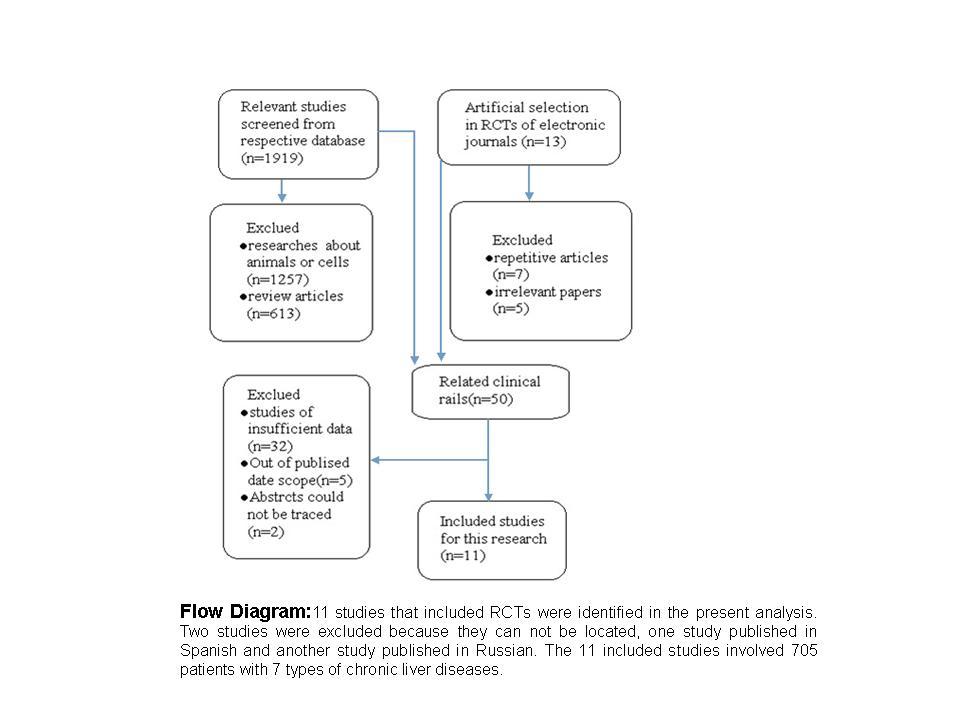

Supplement: S1 Fig — 11 studies that included RCTs were identified in the present analysis. Two studies were excluded because they can not be located, one study published in Spanish and another study published in Russian. The 11 included studies involved 705 patients with 7 types of chronic liver diseases. (JPG) [file pone.0122124.s001.jpg]
